# GP-initiated preconception counselling in a randomised controlled trial does not induce anxiety

**DOI:** 10.1186/1471-2296-7-66

**Published:** 2006-11-03

**Authors:** LC de Jong-Potjer, J Elsinga, S le Cessie, KM van der Pal-de Bruin, A Knuistingh Neven, SE Buitendijk, WJJ Assendelft

**Affiliations:** 1 Department of Public Health and Primary Care, Leiden University Medical Center, Leiden, The Netherlands; 2 Department of Medical Statistics and Bioinformatics, Leiden University Medical Center, The Netherlands; 3 TNO Quality of Life, Division of, Leiden, The Netherlands

## Abstract

**Background:**

Preconception counselling (PCC) can reduce adverse pregnancy outcome by addressing risk factors prior to pregnancy. This study explores whether anxiety is induced in women either by the offer of PCC or by participation with GP-initiated PCC.

**Methods:**

Randomised trial of usual care versus GP-initiated PCC for women aged 18–40, in 54 GP practices in the Netherlands. Women completed the six-item Spielberger State Trait Anxiety Inventory (STAI) before PCC (STAI-1) and after (STAI-2). After pregnancy women completed a STAI focusing on the first trimester of pregnancy (STAI-3).

**Results:**

The mean STAI-1-score (n = 466) was 36.4 (95% CI 35.4 – 37.3). Following PCC there was an average decrease of 3.6 points in anxiety-levels (95% CI, 2.4 – 4.8). Mean scores of the STAI-3 were 38.5 (95% CI 37.7 – 39.3) in the control group (n = 1090) and 38.7 (95% CI 37.9 – 39.5) in the intervention group (n = 1186).

**Conclusion:**

PCC from one's own GP reduced anxiety after participation, without leading to an increase in anxiety among the intervention group during pregnancy. We therefore conclude that GPs can offer PCC to the general population without fear of causing anxiety.

Trial Registration: ISRCTN53942912

## Background

By addressing pregnancy-related risk factors and preventive measures before pregnancy, preconception counselling (PCC) reduces the number of adverse pregnancy outcomes [1]. Currently, PCC is offered almost exclusively to women at known risk of adverse pregnancy outcomes (e.g. women at high risk of thrombosis, or with insulin-dependant diabetes) [2,3].

Little is known about women's perception of PCC, or whether it might have any adverse effects. Various studies in genetic testing have reviewed the effect on anxiety and reported no long term adverse psychological effects. However, these tests concerned people at high-risk of cancer or hereditary diseases[4,5] or high-risk pregnancies[6,7]. Recently a number of studies found no serious psychological harm to low-risk pregnant women after serum screening [8,9] or early ultra-sound examinations[10] to detect an increased risk for Down's syndrome. One study on the other hand, reported an adverse impact on women's perceptions of their own health after screening for gestational diabetes [11] and another found that labelling pregnant women as 'at risk' affected their psychosocial state negatively [12]. Not only is maternal anxiety undesirable during what should be an essentially natural life-event, it has also been associated with adverse pregnancy outcomes [13]. While, in high-risk groups, the potential risks to the foetus outweigh the possible induction of anxiety, PCC for low-risk groups is still controversial, and its pros and cons have not been studied sufficiently. PCC offered at a university fertility clinic did not increase the anxiety levels of women who followed the programme [14]. Even so, knowledge whether PCC induces anxiety in a low-risk population is indispensable before initiating a systematic PCC programme for the general population.

The fact that merely everyone in the Netherlands is registered with a general practice provides an ideal opportunity to reach couples with a low-risk profile at an appropriate time for PCC. As part of an intervention study, 'Parents to Be', we developed a systematic PCC programme specifically designed for GPs to offer to women of childbearing age [15]. The main objective of the trial was to identify whether PCC could reduce adverse pregnancy outcome. To explore whether any anxiety might be created either by the offer of PCC or by participation in it, we measured anxiety levels both before and after a PCC session.

To determine whether the offer of PCC actually induced anxiety during pregnancy, we compared the anxiety levels of a group of women who became pregnant after they had been offered PCC with those of a group who were not.

## Methods

### Parents to Be trial

A total of 110 GPs were approached and 67 GPs of 54 practices agreed to take part in the trial. The participating general practices were divided in strata according to the characteristics both of the practice and of the GPs (age, gender, practice population size, practice situation, percentage ethnic minorities). Within these strata, practices were randomised (by computer) either to the intervention group or to the control group, resulting in 27 practices (30 GPs) in the intervention group and 27 practices (37 GPs) in the control group. Comparable numbers of patients were registered at the intervention and control practices. The power calculation for the main trial was based on an absolute risk reduction of 4% in adverse pregnancy outcome between these two groups of patients. We assumed that 30 pregnancies would occur in a practice each year.

### PCC procedure

Prior to the start of the trial, GPs in the intervention group received training on how to provide PCC. Subsequently, all women 18–40 years of age were selected in the intervention group. Women were excluded if they had undergone hysterectomy or sterilisation, if they were known to be subfertile or infertile, if they had an insufficient knowledge of the Dutch language, if they were difficult social circumstances in their lives or if the GP presumed they had completed their family.

The remaining women received a letter explaining the study procedure and were invited to attend PCC. The selection and the offer of PCC took place in 2000, 2001 and 2002 [16]. Women were asked to indicate if they were interested and if so, when they were planning a pregnancy. Women who were interested in PCC and were planning a pregnancy within one year were sent a questionnaire assessing personal risk factors of the future parents for adverse pregnancy outcomes on the basis of their personal history of acute and chronic diseases, infectious diseases, nutritional and behavioural habits, and also on their family history of genetic diseases. This risk assessment questionnaire was based on the preconceptional health assessment form developed by Cefalo et al[17]. Besides textbooks and recent literature were searched for items that should be added to the risk assessment questionnaire, such as folic acid. Items were only included if the issue was considered amenable to PCC. The resulting questionnaire was reviewed and adapted by a panel of experts including an obstetrician, a paediatrician, a clinical geneticist, a general practitioner and a communications expert for face and content validity. The development and results of the risk assessment questionnaire are described in detail elsewhere. (Elsinga J, de Jong-Potjer LC, van der Pal-de Bruin KM, van Haeringen A, Knuistingh Neven A, Verloove-Vanhorick SP et al.: Preconception counselling in primary care: prevalence of risk factors among couples with pregnancy wish. Submitted 2006)

### Anxiety prior to and following PCC

At the end of the risk assessment questionnaire prior to PCC, women were asked to fill in the Dutch version [18] of the six-item short-form Spielberger State Trait Anxiety Inventory (STAI) [19]. The six-item STAI was prorated to be equivalent to scores obtained using the full form of the scale (α = 0.82), giving a range of 20–80, whereby a low score indicates less anxiety [19]. The mean for a normative sample of women being 35,2 and scores higher than 42 indicate a clinically significant level of anxiety[18]. It consists of statements referring to feeling calm, tense, upset, relaxed, content or worried on a four-point scale. The six-item short-form STAI was recently validated in preconception counselling [20].

Participants were asked a number of questions about their perception of their own risk of having a child with a congenital disorder, and how they judged their own knowledge of preventive measures for such disorders. The questionnaire was subsequently returned to the GP in time to prepare for the counselling session.

During the session, the GP discussed the individual risk factors of both partners on the basis of the risk assessment as well as general risk factors. Issues that were discussed were genetic counselling, obstetric risk factors, infection prevention, medication use, folic acid use, intoxicants (e.g. alcohol and smoking), nutrition and occupational hazards. Immediately after the PCC session, women were asked to fill in another STAI form (STAI-2).

### Anxiety level first trimester of pregnancy

All women in both the control and intervention practices who had been pregnant between April 2000 and April 2003 received a questionnaire two months after their pregnancy had ended. GPs excluded women if taking part in the study was thought to be too burdensome due to emotional problems. Lifestyle and behaviour before and during pregnancy, as well as complications and pregnancy outcome were evaluated. At the end of the questionnaire, women were asked to complete the STAI on the basis of their memory of the first trimester of their pregnancy (STAI-3).

The medical ethics committee of Leiden University Medical Center approved questionnaires and study.

### Statistical analyses

The analyses for STAI-1 and STAI-2 were performed using SPSS 11.5. Risk factors for adverse pregnancy outcomes reported in the risk-assessment questionnaire were divided into three categories. Category I comprised risk factors for which written information would be sufficient, category II comprised risk factors for which personal counselling by the GP was indicated (including possible referral to a specialist), and category III comprised risk factors for which a referral to a specialist was necessary. (Elsinga J, van der Pal-de Bruin KM, de Jong-Potjer LC, Assendelft WJJ and Verloove-Vanhorick SP. Preconception counselling in primary care: prevalence of risk factors among couples contemplating pregnancy. Submitted 2006.)

Using paired t-tests, we compared anxiety levels prior to the PCC session (STAI-1) with levels after PCC (STAI-2). Using one-way analysis of variance, with trend test -if appropriate- we related background characteristics, the women's perception of risk, and the number of risk factors in categories II and III to the baseline STAI and the change in STAI after PCC [21].

The anxiety levels (STAI-3) of women who were offered PCC were compared with those of women not offered PCC, as well as with those women who had accepted the offer and those who had not. A three-level model was used with questionnaire as first level, women as second level and GP-practice as third level to compare STAI-3 between the groups. We chose to do so, because of the required cluster randomisation and due to the fact that some women had been pregnant more than once during the study period. This model was fitted using Proc Mixed in SAS version 9.1.3

Subgroup analyses were performed for age, education, country of origin and adverse pregnancy outcome, the latter being defined as miscarriage, preterm birth, stillbirth or congenital disorder of the newborn.

## Results

### Anxiety levels prior to and following PCC

**Figure 1:**
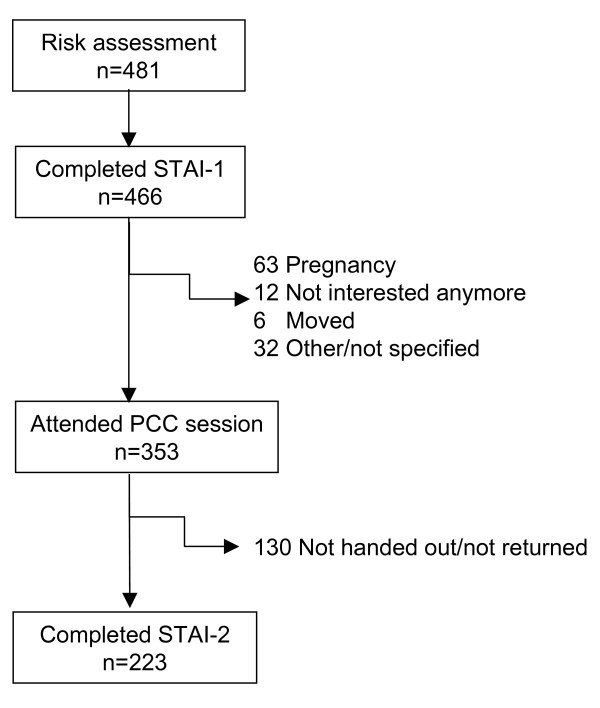
Number of women wishing to conceive and interested in PCC and further participation in PCC. PCC: Preconception counselling session. STAI: 6-item Spielberger State Trait Anxiety score.

In the intervention group, the risk-assessment questionnaire was completed and returned by 481 women, 466 of whom completed the first STAI in full. Participation and response is described in figure 1.

Of the 353 women who actually attended PCC, 223 completed STAI-2 after PCC. The other 130 STAI-2 forms were either not handed out by the GP or were not returned.

The mean STAI-1 score filled in before PCC was 36.4 (95% confidence interval (CI) 35.4 – 37.3), and showed no difference between women who did and who did not actually attend PCC. Table 1 shows the relationship between the mean STAI-1 score and background characteristics.

**Table 1:** Mean STAI of the total group and mean STAI of the group that filled in both STAI-1 and STAI-2; decrease in anxiety score from STAI-1 to STAI-2 relative to background characteristics.

	Total group STAI-1		Group that completed the STAI both prior and post PCC				
		
	n *	STAI	n *	STAI-1 (prior)	STAI-2 (post)	Reduction in STAI score	95% CI
Total	466	36.4	223	36.4	32.8	3.6	[2.4–4.8]
Age groups							
18–25	67	36.9	31	36.9	33.9	3.0	[-0.6–6.7]
26–30	197	35.4	92	36.5	32.4	4.1	[2.0–6.1]
31–35	145	37.1	69	36.5	33.3	3.2	[1.2–5.2]
>36	43	37.4	27	36.0	31.7	4.3	[1.6–7.0]
Education							
University/College	187	35.7 ‡	100	35.5	31.5	4.0	[2.2–5.9]
Intermediate secondary	206	35.9	89	35.5	33.3	2.3	[0.6–4.0]
Basic secondary or less	70	39.2	32	41.1	35.8	5.3	[1.5–9.1]
Country of origin							
The Netherlands	433	36.0 §	210	36.0	32.6	3.4	[2.1–4.6]
Other	32	41.0	13	44.1	36.4	7.7	[1.5–13.9]
Previous pregnancy							
No	310	35.6¶	153	35.6	32.4	3.2	[1.9–4.5]
Yes	155	38.0	70	38.2	33.7	4.5	[2.0–7.1]
No adverse outcome	91	36.7	35	36.0	31.2	4.8	[0.5–9.0]
Adverse outcome†	64	39.8	35	40.4	36.1	4.3	[1.2–7.4]

* Numbers may differ due to non-response. † Miscarriages, stillbirth or premature birth. Test for trend ‡ p-value = 0.04, ANOVA for dichotomous variables § p-value = 0.008, ¶ p-value = 0.02.

Higher baseline scores were found among women with a basic education (39.2) and women of non-Dutch origin (41.0). Women who had been pregnant before had a higher mean STAI-1 score (38.0) than those who had not been pregnant (35.6). This was partially accounted for by the anxiety of the women who had a previous adverse pregnancy outcome (39.8).

Higher anxiety levels were found in women who believed they had a higher risk than others of bearing a child with a congenital disorder (40.6) or those who perceived their subjective risk as high (42.2) (Table 2).

**Table 2:** Mean STAI of the total group and mean STAI for the group that filled in both STAI-1 and STAI-2; decrease in anxiety score from STAI-1 to STAI-2 relative to reported perceptions.

	Total group STAI-1		Group that completed the STAI both prior and post PCC				
		
	n *	Mean STAI	n *	STAI-1 (prior)	STAI-2 (post)	Reduction in STAI score	95% CI Delta
Women's estimation of their own risk of bearing a child with a congenital disorder							
Higher than average	54	40.6†	28	37.4	32.6	4.8	[1.5–8.0]
Average	316	36.4	152	36.9	33.5	3.4	[2.1–4.8]
Lower than average	94	33.7	42	34.1	30.3	3.9	[0.2–7.4]
Subjective perception of height of estimated risk							
High	40	42.2 †	19	40.2	33.5	6.7	[2.8–11.6]
Average	297	36.4	144	36.8	32.6	4.2	[2.6–5.6]
Low	119	33.7	54	33.9	33.0	0.9	[-1.0–3.3]
How many prevention measures women thought were available							
Many	189	35.8	92	35.1	31.6	3.5	[1.6–5.3]
Average	145	37.6	72	37.6	32.5	5.1	[3.0–7.3]
Few	85	34.9	39	35.9	34.1	1.8	[-0.6–4.2]
Do not know	33	36.3	19	38.2	37.0	1.2	[-4.2–6.6]
Estimated level of knowledge of prevention measures							
High	143	34.5‡	59	33.5	31.5	2.0	[-0.3–4.4]
Average	231	36.8	116	37.3	33.4	3.9	[2.3–5.5]
Low	86	38.2	47	38.2	33.0	5.2	[2.2–8.1]
Number of category II risk factors							
0–4	114	33.8 †	55	34.2	30.6	3.6	[1.7–5.4]
5–9	292	36.5	135	36.5	32.6	3.9	[2.3–5.4]
10–14	61	40.2	33	39.9	37.1	2.8	[-1.6–7.3]
Number of category III risk factors							
0	374	36.3	184	36.5	32.6	3.9	[2.7–5.3]
1 or more	93	36.5	39	35.8	33.8	2.0	[-1.1–5.1]

*Numbers may differ due to non-response. Test for trend † p-value < 0.0001, ‡ p-value = 0.006.

Anxiety was significantly increased in women who felt they had little knowledge of preventive measures (test for trend p = 0.006). There was an association between the number of actual risk factors for adverse pregnancy outcome in category II, which required personal counselling, and the mean STAI-1 score (r = 0.19, p < 0.001). No significant correlation was found with category III items, which entailed referral to a specialist (r = 0.02, p = 0.67).

Comparison of anxiety levels prior to PCC with the levels afterwards showed an average decrease of 3.6 points (95% CI, 2.4 – 4.8). No difference was found in the mean STAI-1 scores between women who did or did not fill in the STAI-2. Neither was a correlation found between the time interval between completing the first two questionnaires and the decrease in anxiety score (r = 0.09, p = 0.2). The highest reduction in anxiety scores was found among women with a basic education (5.3, 95% CI 1.5–9.1) and women born in countries outside the Netherlands (7.7, 95% CI 1.5–13.9). Relatively speaking, the reduction in anxiety was also larger in women who estimated that they had a high chance of having a child with a congenital disorder (6.7, 95% CI 1.7–11.6). Women who knew little of preventive measures were initially anxious and showed a substantial decline of 5.2 (95% CI 2.2 – 8.2). After PCC there was a smaller decrease in anxiety in women with the highest number of category II risk factors and in those women with serious (category III) risk factors (2.8 respectively 2.0).

### Anxiety level in first trimester of pregnancy

**Figure 2:**
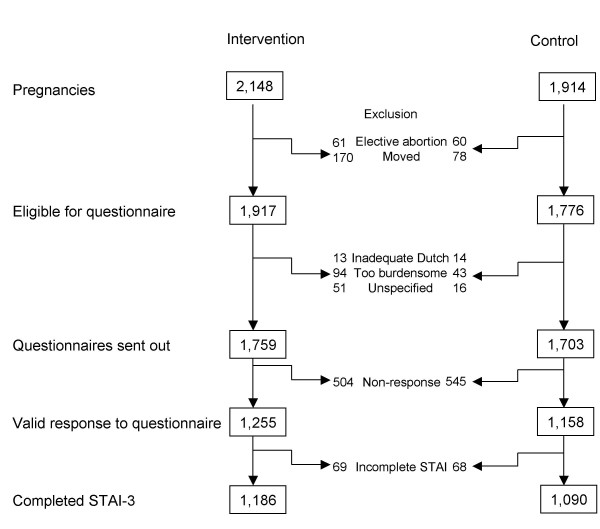
Number of pregnancies, participation post-pregnancy questionnaire, response and completed STAI-3. STAI: 6-item Spielberger State Trait Anxiety score.

During the study, 4,062 pregnancies occurred. Anxiety was measured following pregnancy on the basis of women's memory of the first trimester of their pregnancy (STAI-3). Eligibility, exclusion and response to the post-pregnancy questionnaire is described in figure 2.

In total, 3,406 (84%) women received the questionnaire and 2,413 returned it (response 71%). 2,276 questionnaires completed adequately were analysed; 1,186 from the intervention group and 1,090 from the control group. Although the age distribution was comparable to that of women of childbearing-age in the general population, the respondents had a relatively higher educational level[22].

As shown in Table 3, the mean STAI-3 scores were 38.5 (95% CI 37.7 – 39.3) in the control group and 38.7 (95% CI 37.9 – 39.5) in the intervention group. Overall, analyses of background characteristics showed no significant differences in anxiety levels between the groups, except among women aged 26–30. The control group's mean STAI-3 was 36.9 (95% CI 35.4 – 38.4) compared to 39.6 (95% CI 38.0–41.2) in the intervention group (p = 0.02). When the degree of participation in PCC was further specified in sub-group analyses, women of non-Dutch origin showed a significant difference in mean STAI-3 score. Of these women, those who had received PCC had a higher mean STAI-3 score (59.6, 95% CI 47.6 – 71.6) than those who did not respond to the invitation (40.6, 95% CI 37.3 – 43.9) or those who only filled in the risk assessment questionnaire (23.9, 95% CI 8.5 – 39.2).

**Table 3:** Mean STAI-3 for control and intervention group, mean STAI-3 score relative to background characteristic. Means are estimated using a multi level model

	Control			Intervention							
			
				Only received invitation		Filled in risk assessment		Received PCC		TOTAL Intervention	
						
	n *	STAI		n *	STAI	n *	STAI	n *	STAI	n *	STAI

Total	1090	38.5		962	38.9	71	37.7	153	37.8	1186	38.7
Age groups											
18–25	88	42.1		55	42.1	6	44.4	4	36.7	65	42.0
26–30	226	36.9		156	39.9¶	11	38.2	39	38.6	206	39.6 ¶
31–35	550	38.5		493	38.1	42	36.5	73	36.9	608	37.9
>36	226	38.7		258	39.1	12	38.2	37	39.1	307	39.1
Education											
University/College	396	36.8		352	37.7	37	34.5	68	36.6	457	37.3
Intermediate secondary	420	39.2		397	39.2	25	38.8	67	38.1	489	39.1
Basic secondary or less	242	39.9		190	40.5	8	47.2	16	39.8	214	40.7
Country of origin											
The Netherlands	974	38.1		886	38.7	68	38.5	148	37.1	1102	38.5
Other	116	41.7		76	40.6	3	23.9^#^	5	59.6§	84	41.2
Adverse pregnancy outcome †											
Yes	203	42.3		169	42.1	8	40.1	21	41.6	198	41.9
No	698	37.3		578	38.0	42	37.8	97	37.9	717	38.0
Unknown ‡	189	39.0		215	39.0	21	36.7	35	35.2	271	38.3

*Numbers may differ due to non-response † adverse pregnancy outcome is either miscarriage, preterm, stillbirth, disorder of the newborn ‡ either duration of pregnancy and/or disorder of the new born unknown. ¶ p = 0.02 versus control group ^# ^p = 0.03 versus received only invitation. § p = 0,003 versus only received invitation and p = 0.0003 versus filled in questionnaire.

## Discussions and conclusion

Preconception counselling provided by the participants' GP did not induce anxiety, nor did the invitations to women of childbearing age to participate in PCC resulted in higher pregnancy-related anxiety levels. The women who benefited most from PCC were those of non-Dutch origin with a basic educational level, those perceiving their subjective risk as high or those with a low estimation of their own knowledge of preventive measures: these groups showed a substantial decline in anxiety after the PCC session.

Although we didn't reach the number of participants we needed for conclusions on adverse pregnancy, we believe that the strength of this study lies in the large number of participants from the overall population of women of child-bearing age. Previous studies on genetic testing,[4,5] screening and PCC in hospital setting[14] had the same conclusions, but had a much smaller number of participants, thereby increasing the possibility of false negative findings.

We considered the possibility of selection bias. Our respondents were relatively well-educated compared to the general population. As women could self-select if they were interested, understandably more often women attended PCC who had never been pregnant before or who had experienced an adverse pregnancy outcome. Another bias could be that GPs were able to exclude women if they believed the questionnaire to be too burdensome emotionally. For this reason fewer women with an adverse pregnancy outcome may have received it, thus leading to a selection bias. For example, 9% of all the pregnancies in the study ended in miscarriage (comparable to the national estimate of 10%), but only 6% of the women who completed the STAI-3 had suffered a miscarriage.

There was a difference between the selection of the intervention and control GPs. Probably due to the repetitive selections for the study, the intervention GPs had become more cautious in selecting women, allowing only 46% of the women with an adverse outcome to receive the pregnancy-related questionnaire; with control GPs, the figure was 73%. Of the women who had experienced an adverse outcome, 76% in the intervention group returned the questionnaire versus 70% in the control group. This may have led the actual anxiety scores in the intervention group to be underestimated. However, subgroup-analyses show that there was no difference in mean STAI-3 scores between the women in the intervention and control groups who had had an adverse outcome.

The retrospective nature of the STAI-3 is debatable. However, this assessment gave us the opportunity to study anxiety among women who had declined PCA and had had an adverse pregnancy outcome. Prospective assessment of the STAI-3 in this group of decliners, e.g. in the first trimester could have acted as an extra reminder to adhere to risk-reducing behaviour, which would have biased the results.

Just like Spielberger[23] who found a negative correlation between education and anxiety levels we found higher baseline STAI-scores among women with a basic educational level. High baseline scores were also found among women who had a high number of risk factors, women of non-Dutch origin, those who perceived their subjective risk as high and those who had a low estimation of their own knowledge of preventive measures. Fortunately, these very groups seemed to benefit most from PCC, as they showed a strong decline in anxiety after the PCC session. Only the women with the highest number of category II risk factors, who were justifiably anxious, showed a smaller decline.

A supplementary questionnaire was used for a subgroup of our sample to assess women's satisfaction with PCC. In 2003 a small group of 25 women participating consecutively in ten different practices received it two weeks after attending PCC. As satisfaction with counselling may be influenced by overall satisfaction with the counsellor, this was measured as well[24]. In total, 92% of participants were satisfied with the PCC session, found it informative and felt no increase in anxiety, insecurity about pregnancy or any increase in their fear of complications (data not shown).

Our baseline STAI-score (36.4) was comparable to normative mean STAI score (35.2)[18] and to the mean STAI score measured before PCC at a Dutch university fertility clinic (35.2) [14] and the score to the score found prior to filling in a family history risk-assessment questionnaire (36.7)[25]. In the latter study Qureshi et al[25] found that the mere filling in of the questionnaire had raised anxiety to 39.4, although this dissipated over time. Four of the 25 women who participated in the previously mentioned satisfaction questionnaire reported feeling worried after finishing the risk-assessment questionnaire. Qureshi also found that counselling by a GP lowered anxiety. In addition we found a correlation (r = -0.41; p = 0.04, two-tailed) between the decrease in anxiety after PCC and an increase in overall satisfaction with the GP. While the same Dutch study at the fertility clinic [14] found a small and non-significant increase in anxiety score, our own study detected a significant decline. Being counselled by a familiar physician may be more reassuring. It is even possible that the reduction in anxiety, which was found directly after PCC, is a usual response after a counselling session by the GP [25,26] and is not specifically attributable to the content of PCC.

The only significant difference in STAI-3 was in the age group 25–30 years where the intervention group was slightly more anxious. This effect was not observed in any of the other age groups and could therefore be due to the fact that we performed multiple hypothesis tests.

Among the women who went for PCC, the mean STAI-3 score relating to the first trimester was 37.8, which did not differ much from the first trimester score of 24 women at the fertility-clinic in Nijmegen (35.7) [14]. While the pregnancy outcome in our study does influence the STAI score, the anxiety level in women with an adverse outcome showed no significant difference between those who were offered PCC and those who were not, nor was there a significant difference between the women with different degrees of participation in PCC. The women who declined further participation after filling in the risk assessment did not have a higher anxiety score. Only a small group of four women originating from other countries (one of whom had been pregnant twice) had higher anxiety levels during pregnancy, even though their anxiety did decrease after they had attended the PCC session. Extra attention during PCC seems justified for women born outside the Netherlands. Understanding risks is difficult and cultural differences may entail different attitudes towards pregnancy, different methods of managing risks and hence different anxiety levels.

In the debate on how to provide PCC, some people wish to concentrate efforts on women who have never been pregnant before. This is understandable, as knowledge of preventive measures is mostly transferred during pregnancy (de Jong-Potjer LC, Elsinga J, Le Cessie S, van der Pal-de Bruin KM, Schoorl E, Sneeuw KCA, Verloove-Vanhorick SP and Assendelft WJJ. Knowledge of pregnancy-related risk factors amongst women of childbearing-age: the need for preconception care. Submitted 2006). It may be unwise however: women in our study who had previously been pregnant tended to have higher baseline anxiety levels. If such women were excluded, it would not be possible to address them.

To reach women in time for PCC it is essential to enhance the awareness of both women and physicians to risk factors in everyday life that need adjusting prior to pregnancy. Yet, until PCC is incorporated as a normal part of pregnancy care, extending a (personal) invitation seems necessary. To do so physicians or other health care workers will need (financial) support. As in the Netherlands, registration at a GP's practice, will facilitate the process of reaching women but it is not imperative.

Our study did not show that PCC induced anxiety when it was offered to women in the general population by their own GP. On the contrary: after the counselling session, the anxiety score was lower. There is no evidence that even those women who had had an adverse pregnancy outcome after declining further participation in PCC suffered any anxiety by the offer of PCC. We therefore conclude that GPs can offer PCC to the general population without fear of causing anxiety.

## Competing interests

The author(s) declare that they have no competing interests.

## Authors' contributions

LCJ was involved in the study design, enrolled participants, monitored data collection, analysed data and drafted the manuscript. JE enrolled participants, monitored data collection and revised the draft. SC was involved in the study design, analysed data and prepared the draft manuscript. KMP was involved in the study design, revised the draft and supervised the project. AKN analysed data and prepared the draft manuscript. SEB revised the draft and supervised the project. WJJA analysed data, prepared the draft manuscript and supervised the project. All authors read and approved the final manuscript.

## Pre-publication history

The pre-publication history for this paper can be accessed here:


